# Transforming Growth Factor β/Activin signaling in neurons increases susceptibility to starvation

**DOI:** 10.1371/journal.pone.0187054

**Published:** 2017-10-30

**Authors:** Wen-bin Alfred Chng, Rafael Koch, Xiaoxue Li, Shu Kondo, Emi Nagoshi, Bruno Lemaitre

**Affiliations:** 1 Global Health Institute, School of Life Sciences, EPFL, Lausanne, Switzerland; 2 Department of Genetics and Evolution, Sciences III, University of Geneva, Geneva, Switzerland; 3 Invertebrate Genetics Laboratory, Genetic Strains Research Center, National Institute of Genetics, Mishima, Japan; Biocenter, Universität Würzburg, GERMANY

## Abstract

Animals rely on complex signaling network to mobilize its energy stores during starvation. We have previously shown that the sugar-responsive TGFβ/Activin pathway, activated through the TGFβ ligand Dawdle, plays a central role in shaping the post-prandial digestive competence in the *Drosophila* midgut. Nevertheless, little is known about the TGFβ/Activin signaling in sugar metabolism beyond the midgut. Here, we address the importance of Dawdle (Daw) after carbohydrate ingestion. We found that Daw expression is coupled to dietary glucose through the evolutionarily conserved Mio-Mlx transcriptional complex. In addition, Daw activates the TGFβ/Activin signaling in neuronal populations to regulate triglyceride and glycogen catabolism and energy homeostasis. Loss of those neurons depleted metabolic reserves and rendered flies susceptible to starvation.

## Introduction

In nature, nutritive sugars are both spatially and temporally variable. During feeding or starvation, multicellular organisms need to modulate metabolic and physical activities in order to maintain an overall positive energy balance. Similar to mammals, systemic regulation of the systemic metabolic landscape is maintained by both the insulin and AKH (glucagon equivalent) signaling in *Drosophila melanogaster*. Sugar homeostasis in flies is maintained by a cluster of neurosecretory cells located in the brain, known as insulin-producing cells (IPCs), and by the retrocerebral complex (corpora cardiaca of the ring gland in larvae) [1]. Together, these structures, act through the secretion of insulin-like peptides (dILPs) and Adipokinetic hormone (AKH) respectively, to regulate sugar homeostasis [2,3,4,5,6]. In addition to these endocrinal mediators of sugar homeostasis, several transcription factors, including ChREBP/Mondo-Mlx (Mio-Mlx), and a zinc-finger domain containing protein, Sugarbabe, have also been implicated in the regulation of sugar metabolism [7,8,9]. Larvae deficient in functional Mondo-Mlx are intolerant towards dietary sugars. These animals have high hemolymph trehalose and glucose, and low TAG, implying defects in hemolymph sugar homeostasis and systemic lipogenesis. It was subsequently discovered that the transcription factor Sugarbabe, which is highly induced upon sugar feeding, is one of the secondary transcription factor that is positively regulated by the Mondo-Mlx transcriptional complex. Despite extensive efforts in describing each of these mediators in sugar metabolism, how each of the factor synergizes remained poorly understood. The effects of other nutritive sugars on these pathways are largely unknown.

More recently, we have shown that the TGFβ/Activin signaling pathway plays a central role in shaping the postprandial digestive landscape in the adult *Drosophila* midgut [10]. The TGFβ/Activin pathway is activated by Activin-β (Actβ), Daw, and Myoglianin (Myo), which via the Type-I receptor Baboon (Babo), activate the R-Smad, Smad2 –also known as Smox [11,12,13]. Specifically, the consumption of nutritive sugars stimulates the expression and secretion of the TGFβ ligand, Dawdle (Daw), from the fat body (a tissue functionally analogous to adipose and liver tissue in mammals). Daw then acts in an endocrine manner to activate midgut TGFβ/Activin signaling, culminating in the repression of carbohydrate- and lipid-acting digestive enzymes in the adult *Drosophila* midgut. In addition, several reports have alluded that the TGFβ/Activin pathway, through Daw, also impinges upon systemic insulin signaling by modulating insulin secretion [14,15]. Yet little is known about how the TGFβ/Activin pathway may affect metabolism beyond the midgut.

In this study, we demonstrate the importance of Daw regulation in energy balance. We show that *Daw* induction by nutritive sugar consumption is dependent on the glucose-sensitive Mlx and dFOXO. Further analysis demonstrated that high *Daw* expression and ectopic activation of the TGFβ/Activin pathway, rendered flies susceptible to sugar deprivation and starvation. Activation of the TGFβ/Activin pathway in neurons did not affect glycogen and triacylglyceride (TAG) storage, but accelerated glycogen and TAG mobilization when flies are under starvation. Our study revealed that Daw functions through the brain to affect whole body lipid and glycogen metabolism.

## Results

### Daw induction by nutritive sugars is mediated by Mlx and dFOXO

**Fig 1:**
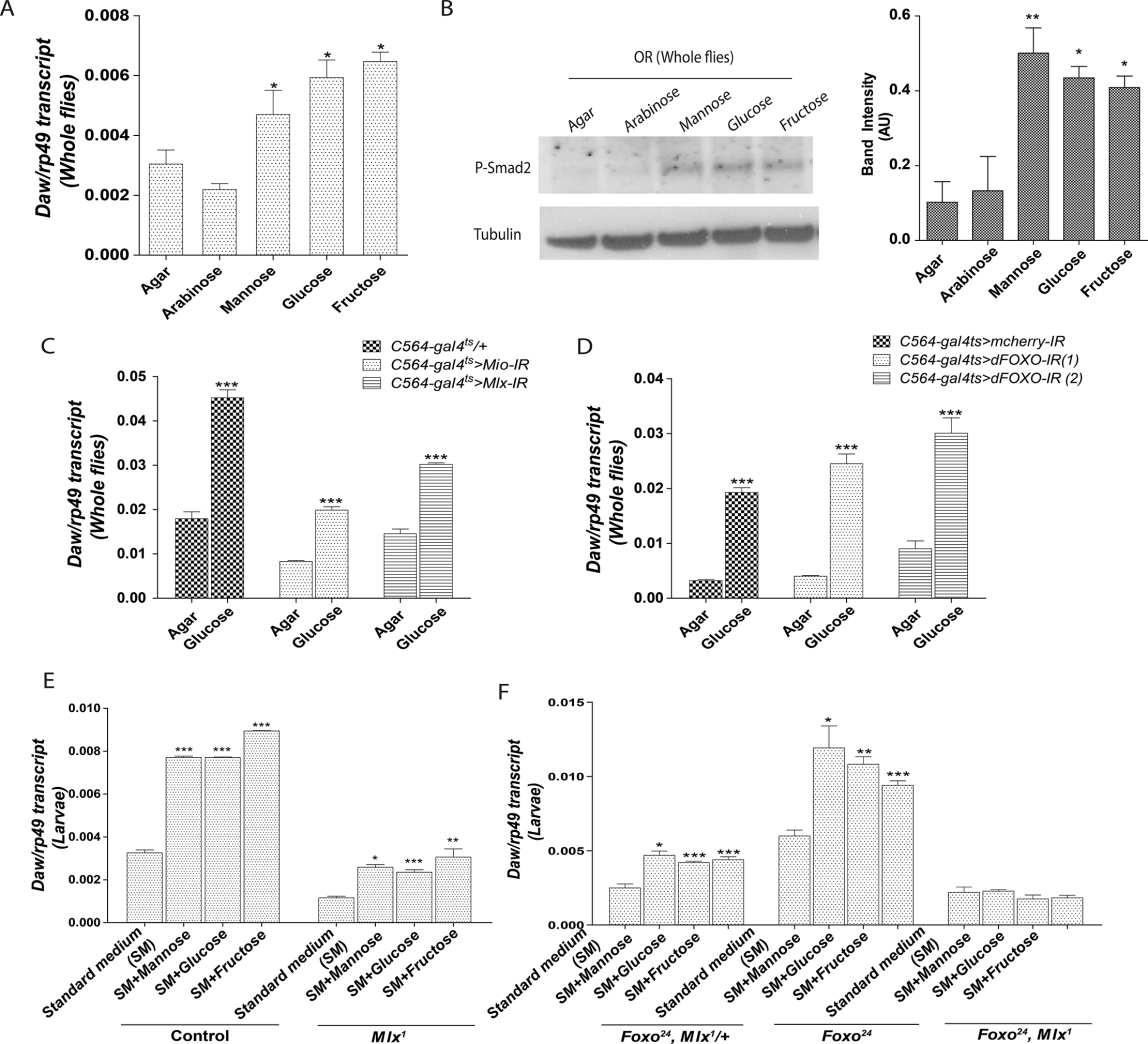
Dietary sugar upregulates *Daw* expression through Mlx and dFOXO. **(A)** RT-qPCR quantification of *Daw* transcript in flies starved on agar or fed on 10% arabinose, 10% mannose, 10% glucose or 10% fructose. All bar graph data represents *Daw* transcript levels, presented relative to *rp49* as mean ±SEM. **(B)** Western blot analysis of P-Smad2 on lysate from flies starved on agar or fed on 10% arabinose, 10% mannose, 10% glucose or 10% fructose. Tubulin is shown as loading control (left panel). Quantification of band intensity (AU: Arbitrary Units), normalized to tubulin is shown on the left (right panel). **(C and D)** RT-qPCR quantification of *Daw* transcript in flies starved on agar or fed on 10% glucose when *Mio*, *Mlx* or dFOXO was knocked down. **(E and F)** RT-qPCR quantification of *Daw* transcript in wild-type, *Mlx*^*1*^ and *Foxo*^*24*^ second instar larvae 48h after egg laying (AEL) on standard medium or standard medium supplemented with 10% mannose, 10% glucose or 10% fructose.

Ingestion of different nutritive sugars leads to the up-regulation of *Daw* transcript [Fig 1A; [10]]. Despite this, it is unclear if transcriptional induction of Daw is indeed translated into an increased in TGFβ/Activin signaling. Activation of the TGFβ/Activin signaling in cells leads to the phosphorylation and activation of the intracellular transcription factor Smad2. Hence, we performed Western Blot analysis on adult wild-type Oregon flies starved on agar, or fed with either a non-nutritive sugar (arabinose) or a nutritive sugar (i.e. glucose, mannose, fructose), and monitored phosphorylated Smad2 levels as a readout for TGFβ/Activin activity. As expected, flies fed with nutritive sugars had higher levels of *Daw* and higher TGFβ/Activin signaling activity compared to flies which were starved, or flies which were fed with arabinose (Fig 1A and 1B; for verification of P-Smad2 antibodies see S1A Fig). Therefore our results confirm that nutritive sugar consumption leads to the induction of *Daw*, and the activation of the TGFβ/Activin signaling pathway.

Next, we examined the underlying mechanism for *Daw* induction in the fat body, a major site of *Daw* expression and induction [10,16]. The transcription factors Mlx (also called Bigmax in *Drosophila*) and dFOXO, are both implicated in the positive and negative regulation of *Daw* expression respectively [8,14]. Although Daw was shown to be regulated by dFOXO and Mlx, it is unclear if glucose-induced Daw expression is mediated through Mlx activity alone, dFOXO alone, or both. Adult knockdown of *Mlx* or its obligate partner *Mio*, using a temperature inducible fat body GAL4 (*C564-gal4*^*ts*^) to drive Mlx or Mio RNAi, reduces *Daw* expression, but failed to abolish *Daw* induction by dietary glucose (Fig 1C). Likewise, adult knockdown of *dFOXO* in the fat body did not abrogate *Daw* induction by glucose (Fig 1D). To ensure that glucose induction is not a result of residual Mlx or dFOXO activity, we tested Daw induction by glucose, fructose or mannose in *Mlx*^*1*^ and *foxo*^*24*^ mutants. Since *Mlx*^*1*^ mutants do not survive to adulthood, analyses of *Daw* induction are examined only in larvae collected 48h after egg laying (AEL) on medium containing different nutritive sugars. Consistent with our RNAi results and data from Mattila et al, (2015), loss of Mlx reduced the expression of *Daw*. However, *Daw* induction by the different nutritive sugars remained (Fig 1E). The ratio of *Daw* induction in *Mlx*^*1*^ was similar to that of a genetically-matched control (S1B Fig). Conversely, the loss of dFOXO function increased *Daw* expression, but did not abolish its induction by nutritive sugars (Fig 1F). Our results led us to hypothesize that nutritive sugars induce *Daw* expression by activating Mlx and repressing dFOXO simultaneously. To test if Mlx and dFOXO are both necessary for *Daw* induction, we examined *Daw* induction by nutritive sugars in *Mlx*^*1*^, *foxo*^*24*^ double mutants. In the absence of both Mlx and dFOXO, *Daw* induction by nutritive sugars is abrogated (Fig 1F). The double mutant larvae display low levels of *Daw*, which is not transcriptionally upregulated by nutritive sugars in the diet. Thus, our results demonstrate that Daw induction by glucose is dependent on Mlx and dFOXO axis of regulation. In addition, it is consistent with a model in which *Daw* induction by nutritive sugar is mediated through the activation of Mlx, which increases *Daw* expression, as well as the concurrent suppression of dFOXO to disinhibit *Daw* expression.

### *Daw* overexpression and ectopic activation of the TGFβ/Activin pathway renders flies susceptible to nutrient limitation independent of Sugarbabe function

**Fig 2:**
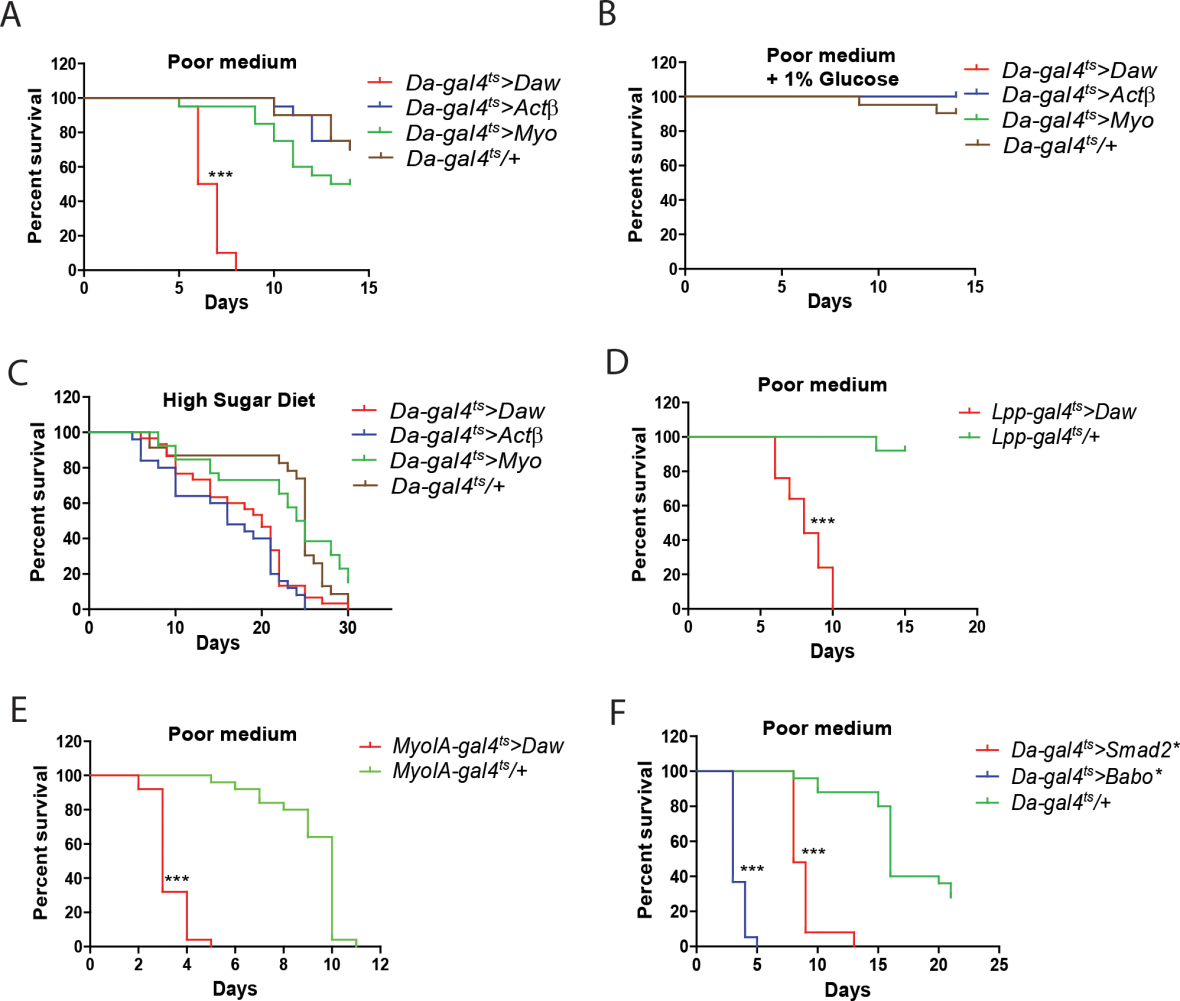
Overexpression of *Daw* and systemic activation of the TGFβ/Activin signaling pathway renders flies sensitive to nutrient limitation. **(A-C)** Survival analysis of females overexpressing different TGFβ/Activin pathway-specific ligands. Flies kept on nutrient poor medium deprived of sugar **(A)**, on poor medium supplemented with 1% glucose **(B)** or on high sugar diet [20% sucrose; **(C)**]. Only the ubiquitous (*Da-gal4*^*ts*^) overexpression of *Daw* was deleterious to flies when raised on a poor diet. **(D-E) Flies over-expressing** Daw in the fat body (*Lpp-gal4*^*ts*^*>Daw*, ***E*)** or midgut (*MyoIA-gal4*^*ts*^*>Daw*, *F)* displayed increased susceptibility when flies were subjected to the poor medium. **(F)** Ubiquitous activation of the TGFβ/Activin pathway by overexpressing constitutively active forms of Babo (Babo*) or Smad2 (Smad2*) increased the susceptibility of flies raised on the poor medium.

The TGFβ ligands, Daw, Myo, and Actβ, utilizes the TGFβ/Activin pathway. However, among the three ligands, only *Daw* is post-prandially induced by dietary glucose [10]. As mutants of these ligands do not survive to adulthood, we employed an overexpression strategy to study their role in adult carbohydrate homeostasis and physiology. To understand the role of Daw in energy homeostasis, we overexpressed Daw, Myo, or Actβ individually using a temperature inducible ubiquitous GAL4 driver (*Da-gal4*^*ts*^) in adults, and subjected female flies to a nutrient limitation protocol. The protocol was performed on a nutritionally poor medium, consisting of 0.62% agar, 0.3% yeast, 1.25% cornmeal, 10.6g/L moldex. Wild-type flies maintained on this poor medium have a prolonged survival (~2 weeks) relative to complete starvation on agar, allowing subtle differences in starvation susceptibility to be studied. Because *Daw*^*1/11*^ mutants were reported to be sensitive to dietary acid [15], propionic acid was excluded from our medium to avoid phenotypes associated with the loss of pH homeostasis. Remarkably, only flies overexpressing *Daw* were maladapted to our poor medium. In contrast, flies overexpressing *Actβ*, or *Myo* had similar survival kinetics to control (Fig 2A). To demonstrate that the survival phenotype is not due to the genetic background of the *UAS-Daw* line, we compared survival of D*a-gal4ts/+* flies with *UAS-Daw/+* flies. As expected, there was no significant difference in susceptibility towards the poor medium (S1C Fig). More importantly, the susceptibility could be rescued by supplementing the poor medium with 1% glucose (Fig 2B), while the survival kinetics of flies overexpressing *Daw*, *Actβ*, *Myo* and control were not significantly different on a high sugar medium (Fig 2C). This indicates that the survival phenotype is not merely differences in lifespan and susceptibility to stress. In addition, consistent with previous reports indicating that Daw functions as an endocrine factor [15], overexpression of *Daw* using either the fat body-specific (*Lpp-gal4*^*ts*^; Fig 2D) or the midgut-specific (*MyoIA-gal4*^*ts*^; Fig 2E) GAL4 driver, both recapitulated the ubiquitous *Daw* overexpression survival phenotype (Fig 2A). All these indicate that the secreted ligand, Daw, plays an important metabolic role distinct from other TGFβ ligands functioning through the TGFβ/Activin pathway.

The receptor-regulated Smad, Smad2, and its upstream receptor Baboon (Babo) are components of the TGFβ/Activin pathway. To demonstrate that the susceptibility phenotype associated with ectopic Daw expression is mediated through the canonical TGFβ/Activin pathway, we overexpressed the constitutively active Babo receptor (Babo*) or the constitutively active Smad2 (Smad2*) ubiquitously, and assessed the survival of those flies on our poor medium. As expected, the ubiquitous activation of the TGFβ/Activin signaling pathway through either Babo* or Smad2* rendered flies more susceptible to the poor medium (Fig 2F and S1D Fig). This susceptibility phenotype was also observed in male flies (S1E Fig). Of note, the overexpression of Babo* appears to be more deleterious than Smad2*. A possible explanation is that Babo* acts on additional downstream pathways in addition to the TGFβ/Activin pathway. Indeed, it was demonstrated that constitutively active Babo also activates the TGFβ/BMP signaling cascade [12]. Therefore, further analysis was performed only with Smad2*. In order to minimize influences from genetic backgrounds, we isogenized the *UAS-Smad2* and *UAS-Daw* element by backcrossing with *w*^*1118*^. The resultant *UAS-Smad2*^w^* and *UAS-Daw*^*w*^ were used thereafter for all further characterization of the TGFβ/Activin pathway and w^1118^ was used as the corresponding control.

**Fig 3:**
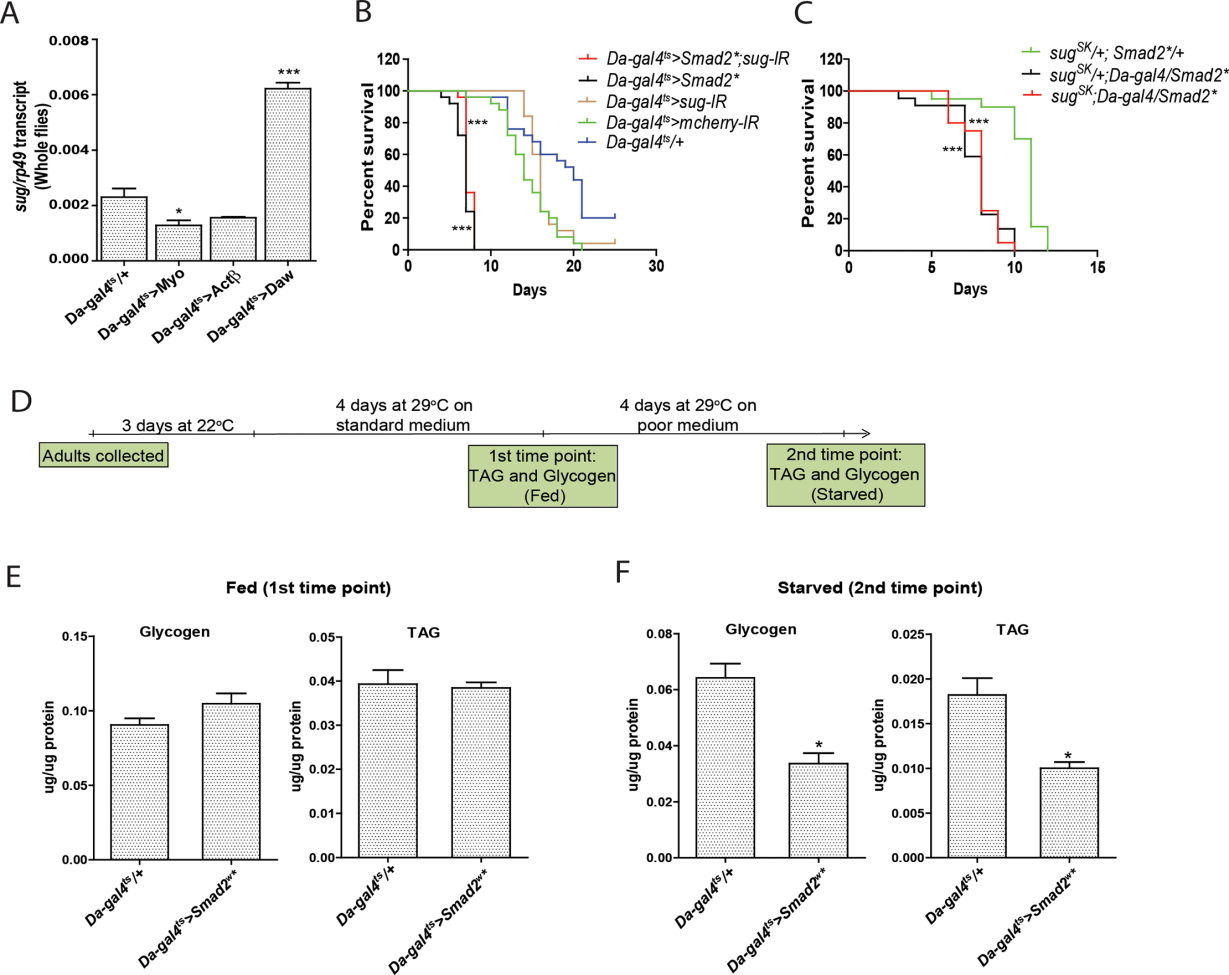
Activation of the TGFβ/Activin pathway enhances glycogen and TAG depletion during starvation. **(A)** RT-qPCR quantification of *sug* transcript in starved flies overexpressing different TGFβ/Activin pathway-specific ligands. Bar graph data is presented relative to *rp49* as mean ±SEM. **(B-C)** Survival analysis of flies with ubiquitous TGFβ/Activin activation and concurrent RNAi-mediated *sug* knockdown, or in Trans with a newly generated viable mutation (*sug*^*SK*^). **(D-F)** Schematic diagram of experimental design (**D**). Glycogen and TAG levels were quantified at two times points and normalized to the level of protein and expressed as μg/μg protein. Flies collected at the first time point were fed on standard medium and had the TGFβ/Activin pathway ubiquitously activated (*Da-gal4*^*ts*^*>Smad2* ^w^*) for four days **(E)**. Flies collected at the second time point were kept four days on the poor medium **(F)**.

A recent study by Mattila and co-workers had shown that the transcription factor Sugarbabe, which is highly induced by dietary sugar [7], is regulated by the TGFβ ligand, Daw [8]. Consistent with their data, we found Sugarbabe expression to be induced by *Daw*, but not *Myo* or *Actβ* overexpression (Fig 3A). However, RNAi-mediated knockdown and loss of function mutation of Sugarbabe (*sug*^*SK*^), which we newly generated, failed to rescue the survival phenotype associated with ubiquitous TGFβ/Activin activation (Fig 3B and 3C). Therefore, the maladaptation survival phenotype associated with TGFβ/Activin activation is independent of Sugarbabe function. Altogether, our results indicate that the transcriptional induction of *Daw* and the activation of canonical TGFβ/Activin pathway is a post-prandial response which must be suppressed during starvation.

### Ectopic activation of TGFβ/Activin signaling in neurons accelerates glycogen and TAG depletion, and renders flies susceptible to starvation

During starvation, glycogen and TAGs are mobilized to sustain essential biological and physical activities. To understand how ectopic TGFβ/Activin activation increases the vulnerability of animals towards nutrient limitation, we quantified key metabolic reserves in whole flies. We collected adult flies at two time points: (i) four days after activating the TGFβ/Activin signaling, and (ii) four days after transfer onto our poor medium. Experimental design and collection time points are provided in Fig 3D. Activation of the TGFβ/Activin signaling did not significantly alter glycogen and TAG levels compared to control flies prior to starvation (Fig 3E). Hence, flies fed on the standard laboratory medium, with or without ectopic TGFβ/Activin signaling activation are metabolically equivalent before starvation ensues. Interestingly, when flies were collected four days after they were transferred onto the poor medium, both glycogen and TAG levels were drastically reduced in flies with ubiquitous and constitutively active TGFβ/Activin signaling (Fig 3F). Hence, the vulnerability of flies to nutrient limitation is likely related to the accelerated depletion of metabolic reserves.

**Fig 4:**
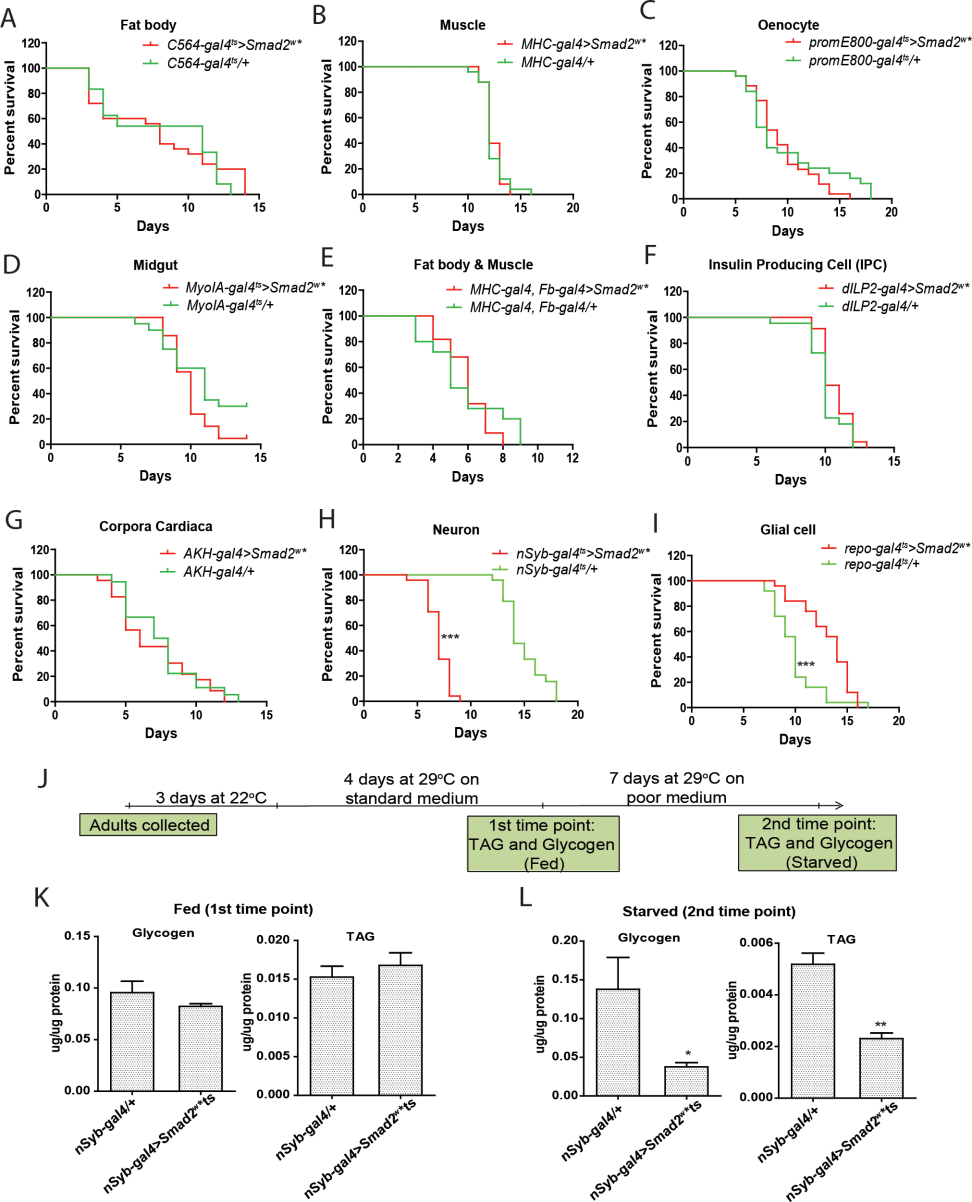
Neuronal activation of the TGFβ/Activin signaling renders flies sensitive to starvation. **(A—I)** Survival analysis of female flies kept on a nutrient poor medium deprived of sugar. The TGFβ/Activin signaling was activated by over-expressing Smad2 in the following tissues: fat body with *C564-gal4*^*ts*^ **(A)**, muscle with *MHC-gal4* **(B)**, oenocytes with *promE800-gal4*^*ts*^ **(C)**, midgut with *MyoIA-gal4*^*ts*^ **(D)**, fat body and muscle with MHC-gal4, Fb-gal4 **(E)**, insulin producing cells with dILP2-gal4 **(F)**, retrocerebral complex with AKH-gal4 **(G)**, neurons with nSyb-gal4^ts^ **(H)**, and Glial cells with repo-gal4 **(I)**. **(J-L)** Schematic diagram of experimental design (**J**). Glycogen and TAG levels were quantified at two times points and normalized to the level of protein and expressed as μg/μg protein. Flies collected at the first time point were fed on standard medium and had the TGFβ/Activin pathway activated in the adult neurons (*nSyb-gal4>Smad2* ^w^*ts) for four days **(K)**. Flies collected at the second time point were kept seven days on the poor medium **(L)**.

Since the fat body is the major site for glycogen and triglyceride storage, we attempted to recapitulate the maladaptation phenotype by activating TGFβ/Activin signaling in the adult fat body (*C564-gal*^*ts*^). However, activation of the TGFβ/Activin pathway in this tissue did not recapitulate the survival phenotype observed with the ubiquitous the GAL4 drivers (Figs 2F and 4A and S1B Fig). Next, to identify Daw responsive tissues underlying the susceptibility phenotype, we expressed the Smad2^w^* using different GAL4 drivers, including those expressed in the insulin producing cells (*dILP2-gal4*), retrocerebral complex cells (*AKH-gal4*), neurons (*nSyb-gal4*^*ts*^), glial cells (*repo-gal4*^*ts*^) midgut (*MyoIA-gal4*^*ts*^), muscle (*MHC-gal4*), oenocytes (*promE800-gal4*^*ts*^), as well as in the muscle and fat body (*MHC-gal4*,*Fb-gal4*). Only the activation TGFβ/Activin signaling in the neurons rendered flies more susceptible to nutrient limitation (Fig 4A–4I). Meanwhile, *nSyb-gal4*^*ts*^*/+* and *UAS-Smad2*^w*^/+ had similar survival profile on the poor medium (S2A Fig). The susceptible phenotype was also observed using an independent pan-neuronal GAL4 (*Elav-gal4*^*ts*^; S1F Fig). This suggested that the suppression of *Daw* expression, and consequently, the reduction of the TGFβ/Activin signaling in neurons, is an important adaptation during periods of nutrient limitation. We then analyzed the effects of neuronal TGFβ/Activin activation on glycogen and TAG stores in flies. Similar to what was observed with ubiquitous Smad2^w^* overexpression (Fig 3E and 3F), overexpression of Smad2^w^* in neurons depleted glycogen and TAG stores only upon starvation (Fig 4J and 4K). In contrast, flies collected prior to the onset of starvation had similar levels of glycogen and TAG. Thus, our data suggest that neuronal activation of the TGFβ/Activin signaling pathway enhances the depletion of energy stores during starvation, and render flies susceptible to starvation.

**Fig 5:**
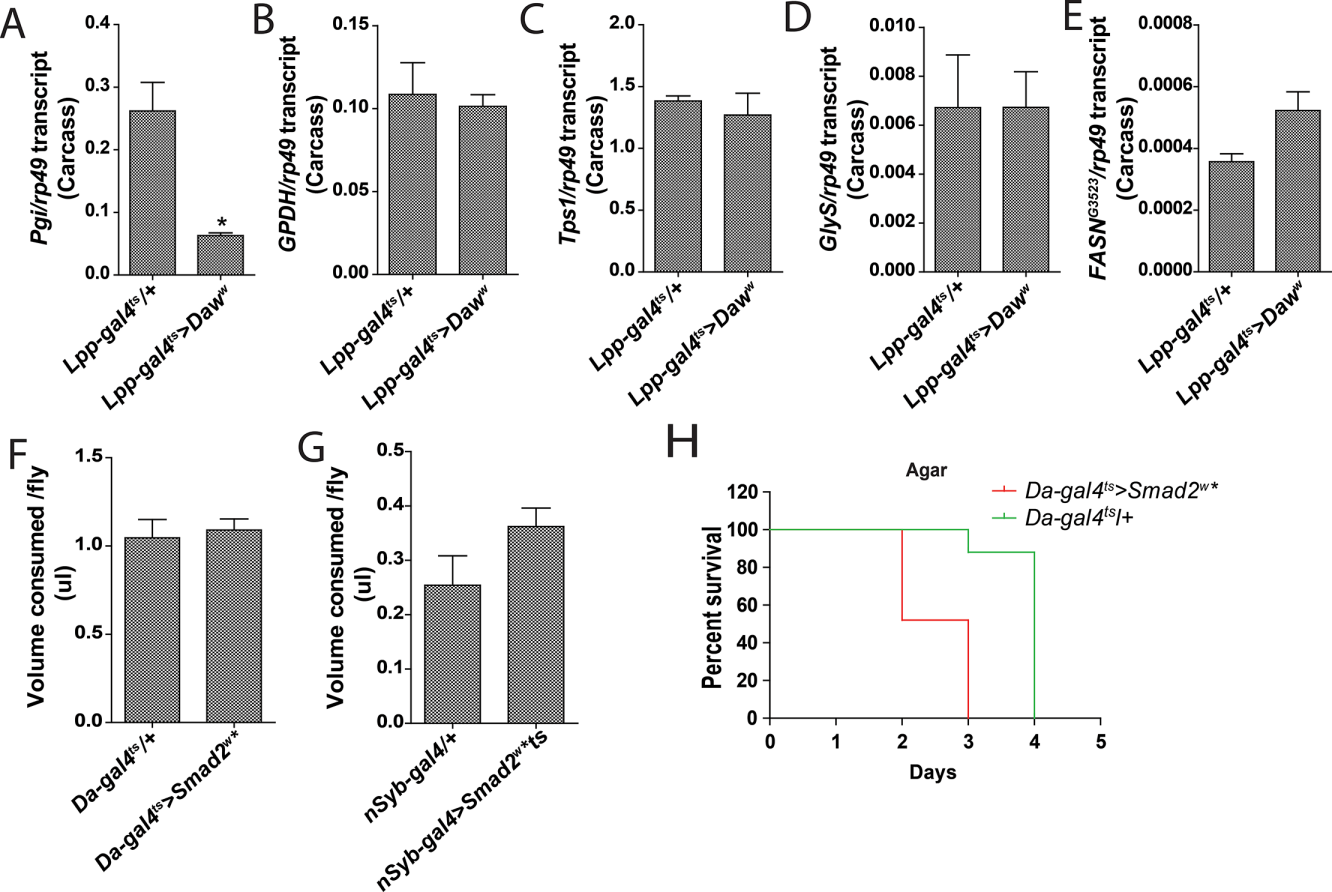
Neuronal TGFβ/Activin activation increases physical activity during starvation. **(A—E)** Expression of genes encoding enzymes involved in various anabolic pathways [glycolysis (**A and B)**, trehalose synthesis (**C)**, glycogenesis (**D**); fatty acid synthesis (**E**)] in carcass derived from starved flies. Data is expressed relative to *rp49* as mean ±SEM. **(F and G)** Food intake is expressed as μl per consumed fly is shown as determined by the CAFE assay. **(H)** Survival analysis of agar-fed flies with ubiquitous TGFβ/Activin activation.

The enhanced rate of glycogen and TAG depletion may result from i) the failure to suppress the expression of counterproductive anabolic pathways, ii) a reduction in food intake and inefficient acquisition of nutrients from the poor medium, or iii) the failure to reduce unnecessary physical activity. To address which of the above contributed the maladaptation phenotype we quantified by qRT-PCR the transcript levels of genes encoding enzymes involved in anabolic pathway that have been shown in several independent transcriptomic analyses to be induced by dietary sugars [7,8,17,18]. We selected a subset for further quantification, reflecting readouts of different metabolic processes. This includes genes involved in glycolysis (*Pgi*, *GPDH)*, trehalose synthesis *(Tps1)*, glycogenesis *(GlyS)* and fatty acid synthesis *(FASN*^*CG3523*^*)*. *Daw* overexpression by the fat body, or ectopic activation of the TGFβ/Activin signaling in the neurons, did not significantly increase the expression of these genes in the fat body relative to control during starvation (Fig 5A–5E, S2B–S2F Fig). In fact, *Pgi* which is repressed during starvation, was much lower in *Lpp-galts>Daw* flies compared with control. This indicates that the transcriptional response to starvation was not overtly affected. Therefore, the failure to repress anabolic genes, does not explain the susceptibility phenotype. Subsequently, we monitored food intake in flies whereby TGFβ/Activin signaling is activated ubiquitously or in the neurons. Neither the ubiquitous nor the neuronal activation of the TGFβ/Activin signaling significantly affected food intake (Fig 5F and 5G). Besides, the susceptibility phenotype persisted when flies were subjected to complete starvation on agar (Fig 5H), which has no utilizable nutrients. Thus, the susceptibility phenotype during starvation cannot be explained by the transcriptional de-repression of counterproductive anabolic pathways, or changes in food intake. During starvation, organisms need to modulate their activity to conserve energy. Considering that ectopic neuronal TGFβ/Activin activation depleted glycogen and TAG stores more rapidly than in control flies, we investigated the impact of neuronal TGFβ/Activin activation on physical activity. Flies were monitored under physiological daily rhythms using the Locomotor activity monitoring system [Trikinetics Inc., [19]]. The system uses infrared beams to detect activity of individual flies trapped in capillaries containing a food source. However, we did not observe any consistent change in physical activity, as measured by the total number of beam crossing events recorded when TGFβ/Activin signaling is activated in the neurons (S2G Fig).

**Fig 6:**
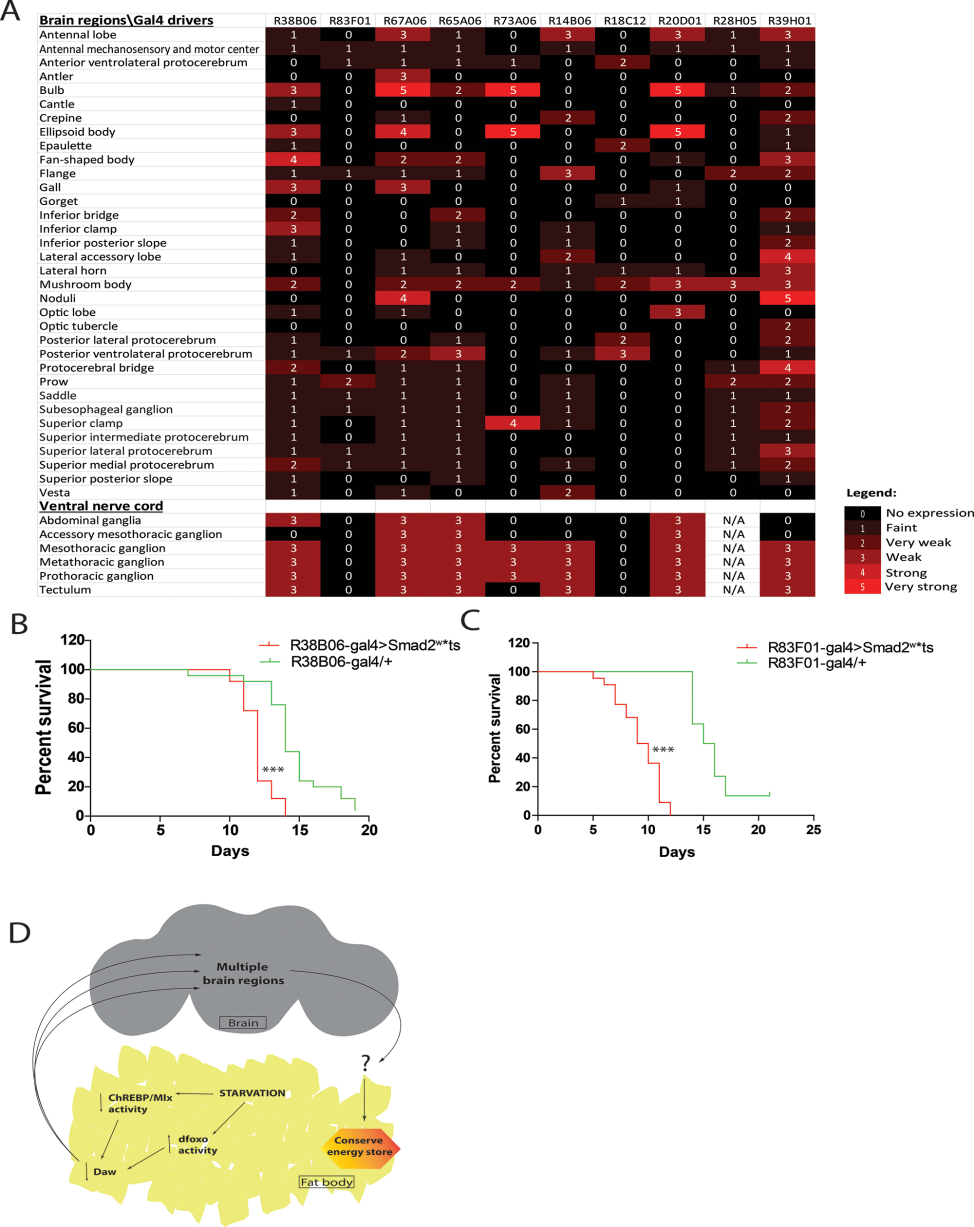
Neurons in different regions of the brain respond when activated rendered flies susceptible to starvation. **(A)** Spatial expression of different neuronal GAL4 drivers used in this study. Expression intensity and region information obtained from Janelia FlyLight Project [20]. **(B and C)** Survival analysis of female flies with specific activation of the TGFβ/Activin pathway in a subset of neuron kept on a nutrient poor medium deprived of sugar. Activation of TGFβ/Activin signaling using two GAL4 lines with expression in different subset both gave rise to flies which were maladapted to starvation. **(D)** A model in which inter-organ communication through Dawdle during starvation. Suppression of Daw through ChREBP/Mondo-Mlx and dFOXO ensures that flies conserve energy stores.

Next, to gain insights into the neuronal populations that respond to TGFβ/Activin signaling, and whose activation increases the susceptibility of flies to our poor medium, we screened a selected panel of partially overlapping neuronal GAL4 drivers. The GAL4 drivers are selected based on their expression in different regions of the adult nervous system [20]. To identify neuronal subsets which play an important role in energy homeostasis, we crossed GAL4 lines to flies carrying *UAS-Smad2*^w^*, and assessed the impact of activating the TGFβ/Activin signaling in the different regions of the adult nervous system on starvation susceptibility. While several neuronal GAL4 lines recapitulated the phenotype observed with the pan-neuronal GAL4 drivers (Fig 4H, Fig 6B and 6C, S1D Fig, S1 Table), two GAL4 lines (*R38B06* and *R83F01*) are expressed with little to no overlaps in the adult nervous system (Fig 6A–6C). Therefore it is likely that the endocrinal Daw may impinge upon multiple neuronal subsets in different regions of the adult nervous system in the adult fly in response to changes in dietary sugar (Model; Fig 6D).

## Discussion

Carbohydrate is an important source of energy, and excess carbohydrate intake is a major contributor to the ongoing obesity epidemic [21,22,23]. Carbohydrate intake, sugar metabolism, and storage are a function of the energy requirements, and studies have shown that physical activity is inversely correlated with obesity [24,25]. In *Drosophila*, hyperactivity has been shown to increase starvation sensitivity. To thrive in an environment that is so often diverse and variable, organisms need to evolve an efficient mechanism to differentiate nutritive carbohydrates from non-nutritive ones (i.e. those that does not provide any caloric value). We have previously described in *Drosophila* how the TGFβ/Activin pathway functions as a signaling entity which relays information about the nutritional value of carbohydrates in the diet. The consumption of nutritive sugars enhances the expression and secretion of Daw, a TGFβ ligand from the fat body, which then functions in an endocrine manner to regulate digestive enzyme expression [10]. Here we define the upstream sugar sensing mechanisms for the transcriptional induction of Daw and describe its role in regulating physical activity.

Intracellular sensing of glucose in the fat body through the ChREBP/Mondo-Mlx complex and activation of insulin signaling by sugar has been previously characterized in *Drosophila* [4,8,26]. Nonetheless, how the two pathways converge and what are their roles in nutritive sugar sensing remained largely unexplored. Here, we show that the induction of *Daw*, an endocrine factor which is induced by nutritive sugar, is downstream of both pathways. Our results showed that loss of function mutation of either dFOXO or Mlx, did not abolish glucose-induced transcriptional induction of *Daw*. Mlx functions as an intracellular sensor for glucose and dFOXO is a well-known target of the glucose-sensitive insulin signaling pathway [4,26]. A recent study has shown that Mlx serves as a positive regulator of *Daw* expression, while dFOXO represses *Daw* expression [8,14]. Little is known about how the two pathways converge. In this study, we have shown for the first time that the induction of *Daw* by different nutritive sugars is dependent on both Mlx and dFOXO. As such, we provided the mechanistic insights for transcriptional induction of *Daw* in the context of nutritive sugar intake and recognition. The integration of both intracellular and extracellular regulation is expected to provide robustness to Daw regulation, which in turn plays an important role in the maintenance of a positive energy balance in response to changes in dietary sugar intake.

Recent studies have strongly suggested that the understudied Daw functions beyond the midgut [10] to regulate different aspects of metabolism. Bai and colleagues have shown that muscle-specific *Daw* knockdown, reduces dILP2 secretion, and increases adult lifespan [14]. In addition, Ghosh and O’Connor have demonstrated the *Daw* mutation affected the TCA cycle flux and pH homeostasis. Moreover, Ghosh and O’Connor observed that *Daw* mutants have a deficiency in insulin secretion and are more susceptible to a high sugar diet [15]; reinforcing the notion that Daw functions as an important endocrine factor for carbohydrate homeostasis. Despite this, little is known with regards to its role in energy homeostasis. There is no study on the role on the physiological role of Daw in adult flies. Here we show that the overexpression of *Daw*, and the activation of the canonical TGFβ/Activin signaling, renders flies susceptible to starvation. The increased susceptibility is independent of Daw midgut function. Although Daw may act upon diverse tissues, the survival phenotype upon starvation appears to be a result of Daw activity on the nervous system. Activation of Daw ubiquitously or in the neurons both led to a higher rate of glycogen and lipid breakdown. This indicates that the flies failed to subdue energy consuming processes which are depleting its energy stores. We were unable to determine what has led to this depletion. Transcript levels of key anabolic genes were not significantly changed, indicating that the susceptibility phenotype is not likely due to transcriptional changes in fat body metabolism, when neuronal TGFβ/Activin signaling was activated. The amount of food consumed by flies with or without TGFβ/Activin signaling activation in the neurons was also similar. Similarly we did not see any meaningful change in physical activity when TGFβ/Activin signaling was activated.

Through further analysis of different neuronal GAL4 driver lines, expressed in a partially overlapping manner, our results demonstrated that activation of TGFβ/Activin signaling in different subsets of neurons had also led to a similar susceptibility phenotype. While it is possible that the two GAL4 lines (R3B06 and R83F01) could also be expressed in the peripheral tissues, we believed that the survival phenotype is mediated through its expression in the neurons. In support of this, all pan-neuronal GAL4 drivers had the same survival phenotype, while the different peripheral tissues drivers tested did not. Interestingly, we noted that activating the TGFβ/Activin signaling in glial cells increased resistance to starvation, indicating an added layer of complexity to glucose-induced Daw response. A key question for future study would be to understand secondary factors are regulated in neurons and glial cells. Altogether, our study demonstrates the importance of repressing *Daw* expression in order to conserve energy during starvation. Future studies on the neuronal subsets identified in this study have the potential to unravel novel targets regulating energy expenditure, which may be exploited against excessive caloric intake and the current obesity pandemic.

## Materials and methods

### Fly stocks

Oregon (OR) flies were used as wild-type flies in experiments to determine TGFβ/Activin signaling activity. The following fly lines were also used in this study: *C564-gal4*^*ts*^ *[10]*. *Da-gal4*^*ts*^: *hs-hid; tub-gal80*^*ts*^*; da-gal4 Dpt2*.*2-lacZ*. Smad2^w^*ts: UAS-Smad2^w*^ts; *UAS-GFP*, *tub-Gal80ts*. *UAS-Actβ* [12]. *UAS-Daw* [27]. *UAS-Myo* [11]. UAS-Smad2* (*UAS-dSmad2-SDVD*). UAS-Babo* [28]. *MyoIA-gal4*^*ts*^: *MyoIA-Gal4*, *UAS-GFP*, *tub-Gal80ts* (from Huaqi Jiang). *AKH-gal4* (Bloomington Center #25684). *MHC-gal4* (Bloomington Center #38464). *promE800-gal4*^*ts*^ [29]. *dILP2-gal4* [5]. *dILP3-gal4* [30]. foxo^24^ [31]. Mlx-control and *Mlx*^*1*^ [9]. *UAS-Smad2*^w^* and *UAS-Daw*^*w*^ was obtained by back crossing with *w*^*1118*^ for six generations.

Sug mutant flies were generated as described in [32] with the guide RNA sequence:5’ -GCGGGGAACTCCACTGGACTG -3’. RNAi lines used were: *UAS-dFOXO-IR(1)* (Bloomington Center # 25997). *UAS-dFOXO-IR(2)* (Bloomington Center # 27656). *UAS-Mlx-IR* (VDRC #110630). *UAS-Mio-IR* (VDRC #109821). As controls, *w*^*1118*^ (Bloomington Center #5905 or VDRC #60000), *yw*, and y[1] sc[*] v[1]; P{y[+t7.*7] v[+t1*.*8] = VALIUM20-mCherry}attP2* (Bloomington Center #35785) *y*,*w[1118];P{attP*,*y[+]*,*w[3`]}* (VDRC #60100) were used in crosses. F1 flies were raised at 18°C or 22°C and switched to 29°C 2–3 days after eclosion.

### Food preparation and diet

All stocks were reared on standard medium consisting of 6% cornmeal, 6% yeast, 0.62% agar, 0.1% fruit juice, supplemented with 10.6g/L moldex and 4.9ml/L propionic acid. Nutritive/Non-nutritive sugar induction in adults is conducted using medium contained 10% glucose, 10% mannose, 10% fructose or 10% arabinose (Sigma) and 0.62% agar, supplemented with 10.6g/L moldex. Complete starvation is performed using agar supplemented with 10.6g/L moldex. Poor medium used in survival experiments consist of 0.62% agar, 0.3% yeast, 1.25% cornmeal, 10.6g/L moldex. High sugar diet consists of standard medium components (excluding propionic acid) supplemented with 20% sucrose. For the study of *Daw* induction in larvae, standard medium (without propionic acid), or standard medium supplemented with 10% glucose, 10% mannose, or 10% fructose were used.

### RNA extraction and RT-qPCR

Total RNA was extracted from 8–10 whole flies or 15 to 20 larvae with Trizol (Invitrogen). 400-500ng of total RNA was used to generate cDNA by reverse transcription (TAKARA, Cat: RR037A) according to manufacturer’s instruction. RT-qPCR was performed by mixing cDNA samples diluted 10-fold for whole flies, and 20-fold for tissue samples, with LightCycler FastStart DNA Master SYBR Green I (Roche) and corresponding primers in a 96-well plate. Expression values were normalized to rp49 and relative quantification was done using the 2^ΔΔCt^ method. At least 3 independent experiments were performed.

### Western blot analysis

Protein samples corresponding to 15 whole flies were prepared with RIPA buffer, supplemented with protease inhibitor cocktail (Roche). 50μg of total lysate as determined by Bradford assay (Sigma) was loaded and separated on a 10% acrylamide precast Novex gel (Invitrogen) under reducing conditions and transferred onto nitrocellulose membrane. Primary antibodies were incubated at 4°C overnight. Subsequently, species-specific HRP-conjugated secondary antibodies were used. Primary antibodies used are as follows: rabbit anti-P-Smad2 (Cell Signaling Technology, #3108) 1:1000; mouse anti-β-tubulin antibody 1:10 000 (Cell Signaling Technology, #4054). Secondary antibodies used were: goat anti-rabbit -HRP 1:5000 (Dako); anti-mouse-HRP 1:5000 (GE Healthcare). Blots were visualized by chemiluminescence using ECL (GE Healthcare) or Chemiluminescent Peroxidase Substrate-1 (Sigma).

### Metabolic measurements

For TAG and glycogen, 5 adult female flies were homogenized in 150 μl PBS containing 0.1% Tween and immediately incubated at 80°C for 10 minutes. Lysates used for TAG and glycogen assays were not centrifuged. TAG and glycogen assays were conducted as described by Tennessen et al., 2014. Samples were assayed using the Tecan Infinite M200 microplate spectrophotometer. TAG and Glycogen levels were determined from a standard curve. Amounts were normalized to protein level, determined by Bradford assay. All experiments were conducted in duplicates or triplicates. At least 3 independent experiments were performed.

### Activity measurements

Flies were grown at 22°C. Eclosed 1–2 day old flies were kept 3 more days at 22°C on standard food to ensure full adult development. Flies were entrained in a 12h:12h Light-Dark (LD) cycle, followed by 3 days at 29°C in identical LD conditions. On the third day, males were separated and transferred into behavior tubes with either 5% sucrose or 2% agar only without added nutrients, and assayed for locomotor behavior at 29°C in constant darkness (DD) using Drosophila Activity Monitors (Trikinetics Inc, USA). Data was binned to 30 minute intervals and analyzed using FaasX software (Picot et al, 2007) and Prism software (Graphpad Software, USA). We chose to analyze locomotor activity in the first full day of DD after a 12h recovery period from anesthesia and transfer to the behavior tubes. Female flies were excluded because oviposition affects locomotor activity, and crawling larvae in the tube interfere with fly activity detection.

### Statistical analysis

Before performing any parametric test, we used Levene’s test to test for equality of variances, and the Shapiro-Wilk test to test for normality. For pairwise comparisons, we used Student’s t-test on the transformed data. ANOVA, followed by Dunnett’s test were used when comparing more than two groups. Pooled data (n≥3) were expressed as means ± standard error of the mean (SEM). p < 0.0005 (***); p < 0.005 (**); p <0.05 (*).

## Supporting information

S1 Fig Sugar-induced TGFβ/Activin signaling is deleterious to flies during starvation. **(A)** Western blot analysis of P-Smad2 on lysates of the adult carcass derived from flies starved on agar or fed on 5% glucose, and lysates derived from wide-type (OR) and Smad2 mutant (Smad2F4) larvae. Tubulin is shown as loading control at the bottom. Image captured on Azure Biosystem.
**(B)** RT-qPCR quantification of *Daw* transcript in flies starved on agar or fed on 10% arabinose, 10% mannose, 10% glucose or 10% fructose. All bar graph data represents *Daw* transcript levels, presented relative to flies fed on standard medium as mean ±SEM.
**(C)** Survival analysis of *Da-gal4*^*ts*^*/+* and *UAS-Daw/+* female flies on poor diet.
**(D-E)** Survival analysis of flies with ubiquitous activation of TGFβ/Activin activation and fed on poor diet **(B; female and C; male)**.
**(F)** Survival analysis of female flies on poor diet with activation of TGFβ/Activin activation in neurons.
(TIF)

S2 Fig The susceptibility to starvation phenotype induced by neuronal TGFβ/Activin activation is not caused by aberrant anabolic gene expression or reduced food intake. **(A)** Survival analysis of *nSyb-gal4*^*ts*^*/+* and *UAS-Smad2*^w^*/+ female flies on poor diet.
**(B—F)** Expression of anabolic genes (glycolysis, A and B; trehalose synthesis, C; glycogenesis, D; fatty acid synthesis, E) in carcass derived from starved flies. Data is expressed relative to rp49 as mean ±SEM. **(G)** Locomotor activity in male flies with TGFβ/Activin activation in adult neurons. Flies were synchronized to light-dark cycles and transferred to behavior tubes with either 2% agar or 5% sucrose. Free-running locomotor activity was assayed for 24h under constant darkness 12h after transfer to behavior tubes.
(TIF)

S1 Table Survival analysis for activation of the TGFβ/Activin signaling with different neuronal GAL4. (XLSX)
