# Supplementary figures and images for: Transforming Growth Factor β/Activin signaling in neurons increases susceptibility to starvation
Source: PLoS One. 2017 Oct 30;12(10):e0187054. doi: 10.1371/journal.pone.0187054 (PMC5662222; doi:10.1371/journal.pone.0187054)

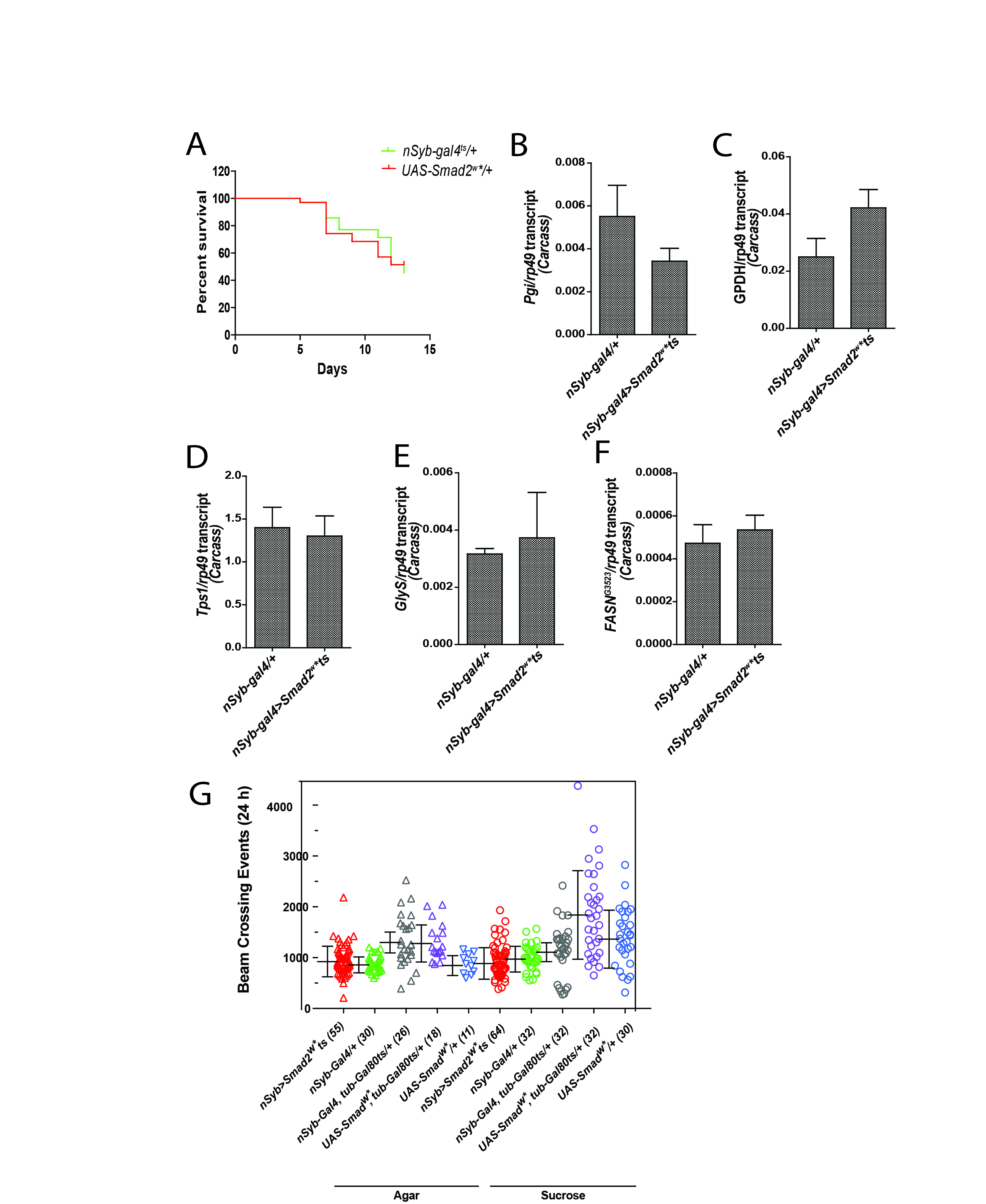

Supplement: S2 Fig — (A) Survival analysis of nSyb-gal4ts/+ and UAS-Smad2w*/+ female flies on poor diet. (B—F) Expression of anabolic genes (glycolysis, A and B; trehalose synthesis, C; glycogenesis, D; fatty acid synthesis, E) in carcass derived from starved flies. Data is expressed relative to rp49 as mean ±SEM. (G) Locomotor activity in male flies with TGFβ/Activin activation in adult neurons. Flies were synchronized to light-dark cycles and transferred to behavior tubes with either 2% agar or 5% sucrose. Free-running locomotor activity was assayed for 24h under constant darkness 12h after transfer to behavior tubes. (TIF) [file pone.0187054.s002.tif]
